# Synthesizing AND gate minigene circuits based on CRISPReader for identification of bladder cancer cells

**DOI:** 10.1038/s41467-020-19314-7

**Published:** 2020-10-30

**Authors:** Yuchen Liu, Weiren Huang, Zhiming Cai

**Affiliations:** 1 National and Local Joint Engineering Laboratory of Medical Synthetic Biology, Institute of Translational Medicine, Shenzhen Second People’s Hospital, The First Affiliated Hospital of Shenzhen University Health Science Center, 518035 Shenzhen, China; 2 Key Laboratory of Medical Reprogramming Technology, Shenzhen Second People’s Hospital, The First Affiliated Hospital of Shenzhen University Health Science Center, 518035 Shenzhen, China; 3 Guangdong Key Laboratory of Systems Biology and Synthetic Biology for Urogenital Tumors, Shenzhen Second People’s Hospital, The First Affiliated Hospital of Shenzhen University Health Science Center, 518035 Shenzhen, China

**Keywords:** CRISPR-Cas9 genome editing, Synthetic biology, Cancer

## Abstract

The logical AND gate gene circuit based on the CRISPR-Cas9 system can distinguish bladder cancer cells from normal bladder epithelial cells. However, the layered artificial gene circuits have the problems of high complexity, difficulty in accurately predicting the behavior, and excessive redundancy, which cannot be applied to clinical translation. Here, we construct minigene circuits based on the CRISPReader, a technology used to control promoter-less gene expression in a robust manner. The minigene circuits significantly induce robust gene expression output in bladder cancer cells, but have nearly undetectable gene expression in normal bladder epithelial cells. The minigene circuits show a higher capability for cancer identification and intervention when compared with traditional gene circuits, and could be used for in vivo cancer gene therapy using the all-in-one AAV vector. This approach expands the design ideas and concepts of gene circuits in medical synthetic biology.

## Abstract (web summary)

Synthetic biology logic gates can be used to distinguish healthy cells from cancer cells. Here the authors design minigene circuits that show more robust identification of cancer cells compared to traditional genetic circuits.

## Introduction

Synthetic biology is an emerging field that reconstructs existing living systems and builds genetic parts, circuits, and systems for application purposes^1,2^. Artificially designed gene circuits sense, integrate, and process molecular signals in living cells and automatically perform preset biological functions^3,4^. The use of gene circuits that sense, record, and respond to in vivo disease signals in medical research has special advantages, which can help the rapid diagnoses and efficient treatments of metabolic diseases and malignant tumors^5,6^. Traditional gene therapies often only introduce a single wild-type gene to compensate for a functional defect in a cell. Artificial gene circuits can perform specific biological functions to intelligently diagnose and treat diseases. For example, the benign and malignant status of cells can be identified according to the expression pattern of a group of oncogenic signaling factors^7^, but not according to expression of the individual genes within that group. One group capitalized on such gene expression patterns by developing a microRNA-based gene circuit, delivered by adeno-associated virus (AAV), which specifically targeted tumor cells^8^.

Bladder cancer is the most common malignant tumor of the urinary system, which mainly occurs in the transitional epithelium of the bladder. Despite a variety of clinical treatments, the 5-year survival rate of bladder cancer patients (about 47% for high-grade type) has not been significantly improved since 1985, and the incidence rates have been increasing every year^9^. In recent years, there has been a trend of younger patients, with increasing treatment costs. The traditional treatment of bladder cancer mainly includes surgery and chemotherapy^10^. Surgery may not completely remove all tumors, so it is easy to have postoperative recurrence. Chemotherapy can damage both cancer and normal cells, resulting in serious side effects in patients. There is an urgent need to develop an efficient and safe treatment for bladder cancer. Clinically, drugs for treating bladder cancer are perfused through the urethra rather than into the blood, and observation and sampling for treatment trials through a cystoscopy exam are convenient. Therefore, bladder cancer is an ideal model to test the therapeutic effect of the synthetic gene circuits delivered by gene therapy vectors, and it is also possible to provide a biological strategy for treating this disease specifically and efficiently.

A key obstacle in creating complex gene circuits is the lack of effective gene regulation tools. The RNA-mediated CRISPR system has the potential to edit nucleic acid sequences and regulate gene expression, thereby providing an efficient tool for constructing gene circuits^11,12^. Based on the CRISPR-mediated logic AND gate circuit constructed by our laboratory, the oncogenic signals in tumor cells were systematically sensed and redirected to activate tumor suppressor genes and initiate the apoptosis process of bladder cancer^13,14^.

Although research of artificial gene circuits has made some progress, it also has some limitations. It is still difficult to achieve robust regulation of multilayer gene circuits that perform functions stably in a complex in vivo environment^15,16^ In the process of designing the gene circuit, researchers should fully consider the regulation relationship among the gene modules that constitute the circuit. They need to use the modular principle to design the modules and achieve quantitative prediction and analyses of the function and behavior of the gene circuit.

However, it is difficult to solve the above core problems completely. For one thing, the intracellular expression of each component of the gene circuit still depends on the classical gene expression mode. The organism mainly “reads” the genetic code stored in DNA through “transcription” and “translation.” These processes are regulated by promoters such as the 5′-untranslated (5ʹ-UTR) regions and other cis-elements. Such elements usually play a key role in gene expression, which in turn controls artificial gene circuits to perform their biological functions. However, gene regulatory elements contain various types of non-conserved motifs. As the relationship between sequence information and regulatory functions is not fully characterized, there are often unexpected interactions with host components^17,18^. At the same time, due to the large coding size of the complex gene circuit, it is difficult to integrate it into the AAV and other therapeutic vectors that have promising clinical applications^19^. The above-mentioned problems severely hinder the translation and application of medical synthetic biological techniques represented by artificial gene circuits, when used in the clinic. The reconstruction of gene expression patterns through DNA code reading technology, therefore, represents a key step in designing artificial gene circuits from scratch.

With the continuous optimization of the ability of the CRISPR system to bind and activate target DNA transcription, artificial gene expression regulation devices based on CRISPR have been widely studied^20^. Recently, we have developed a CRISPR reader (CRISPReader)^21^, which involves technology used to control the expression of promoter-less genes in a robust way. With this technology, a positive feedback expression loop was implemented through preliminary transcription initiation relying on the TATA box and subsequent promotion of transcription using the sgRNA-mediated binding of dCas9-based transcriptional activators to the upstream sequence of the TATA box. This tool is very effective in controlling gene expression, and can read the open reading frames of gene clusters without traditional regulatory elements or other cofactors. We assumed that the CRISPReader could simplify over-redundant gene circuits, eliminate the mutual interference between regulatory elements and the cell genome, and achieve all-in-one delivery of the gene circuit through the AAV vector.

In this work, we construct AND gate minigene circuits based on the CRISPReader, and then test the ability of these circuits to selectively identify bladder cancer cells. We show that the highly controlled minigene circuits have much better cancer diagnosis efficiency than that of the traditional logic AND gene circuits we previously constructed^14^. This study, therefore, provides an application tool for bladder cancer, and may provide a potentially effective solution to the current obstacles associated with the use of medical synthetic biology.

## Results

### Design, construction, and test of minigene circuits

**Fig. 1:**
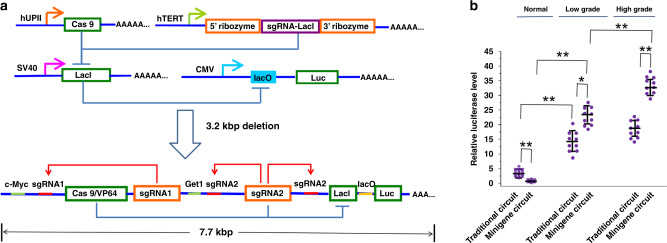
Design and construction of the AND gate minigene circuits. **a** The UPII promoter drove the transcription of Cas9 mRNA, while the TERT promoter was used to promote the transcription of sgRNA targeting LacI. The output Renilla luciferase gene was regulated by a LacI-controlled CMV promoter. The luciferase was expressed only when both UPII promoter and TERT promoter were both activated. In the design of the minigene circuit, the UPII and TERT promoters were replaced by their respective transcription factor binding elements. Both c-Myc and Get1, only in bladder cancer cells, had a relative high expression level at the same time. After initial expression of sgRNA1 and sgRNA2, they could further bind upstream of their own transcription initiation sites and amplify the transcription signals of c-Myc and Get1 through the positive feedback mechanism to amplify their downstream genes transcription, respectively. Furthermore, the LacI gene was knocked out by sgRNA2, and luciferase reporter gene was activated by transcription. In normal bladder epithelial cells, luciferase could not be effectively transcribed and was further silenced by trace amounts of LacI expressed at the background level. **b** The traditional gene circuit and the minigene circuit were compared by detecting the expression levels of luciferase in different bladder cancer cells and normal bladder cells, and the cancer diagnostic efficacy was analyzed. All transient transfections were performed in the presence of PGL3-TK-Fluc vector. Each experiment was independently performed in triplicate five times. The small purple dots represent the relative luciferase level (Rluc/Fluc expression ratio) of each cell line, while the black lines show mean ± SD. ^*^p < 0.05 and ^**^p < 0.01 between the groups using ANOVA. Exact *p*-values for asterisks (from left to right): 0.0038, 0.0045, 0.0018, 0.033, 0.0045, and 0.0025. Source data are provided as a Source Data file.

In previous work, we constructed an AND gate based on CRISPR-Cas9 that specifically targeted bladder cancer cells (Fig. 1a). This circuit used a bladder cell-specific promoter of the human uroplakin II gene (hUP II) and tumor cell-specific promoter of human telomerase reverse transcriptase (hTERT) as biosensors in response to endogenous transcription signals in cells. The hUP II promoter drove the transcription of the Cas9 gene, and the hTERT promoter controlled the expression of sgRNA. The repressor LacI protein inhibited the transcriptional initiation of downstream effector genes by binding to an operon sequence, and the sgRNA-Cas9 system mediated knockout of the *LacI* gene. Our previous results suggested that only in bladder cancer cells could the two input promoters be simultaneously activated to drive downstream output gene expression effectively.

Although the logical AND gate gene circuit could distinguish bladder cancer cells from normal bladder epithelial cells, it still needed to be further characterized. Bladder cancer tissues of different grades and stages and normal tissues adjacent to the cancer were recently collected by our group (Supplementary Table 1). Twenty bladder cancer cell lines with different malignant degrees and corresponding normal bladder epithelial cell lines were separated and cultured to further test the output luciferase expression controlled by this gene circuit (Fig. 1b). Among them, four high malignant cell lines, four low malignant cell lines, and three normal cell lines with good growth were used for follow-up cell functional experiments. The expression level of the luciferase reporter gene was detected (Fig. 1b), and it was found that the expression level of luciferase in bladder cancer cells was 7–8 times higher than that in normal cells, but there was no significant difference in the expressions of luciferase between the subpopulations of bladder cancer cells with high and low malignant potentials. There were also detectable levels of luciferase expression leakage in normal cells.

To optimize the tumor diagnosis efficiency of this circuit further, we used the CRISPReader technology to simplify the circuit design by removing promoters, enhancers, and 5′-UTR (Fig. 1c). First, Cas9-VP64 was introduced to the gene circuit because the action mode of Cas9-VP64 could be switched between the transcriptional activation and DNA cleavage by adjusting the length of the sgRNA spacer, as described in our previous work on CRISPReader^21^ and another earlier study^20^. Cas9-VP64 induced DNA cleavage when the sgRNA spacer was 20 nt in length, while Cas9-VP64 only exerted a transcriptional activation effect when the sgRNA spacer was 14 nt in length. Moreover, the targeting specificity of Cas9-VP64 is better than that of Cas9 protein. Then, the UPII promoter and TERT promoter elements were deleted, and only the corresponding transcription factor binding element was retained. It has been confirmed in previous studies that the tumor targeting transcription activity of the TERT promoter sequence is mainly determined by the c-Myc transcription factor^22^, while the specificity of the bladder epithelial tissue of the UPII promoter sequence is mainly regulated by the Get1 transcription factor^23^. In theory, both c-Myc and Get1 should have a relative high expression level only in bladder cancer cells. After initial expression, the sgRNAs can further bind upstream of their own transcription initiation sites (TATA boxes) and amplify the activation effects of the transcription signals (c-Myc and Get1) through a positive feedback mechanism to drive their downstream gene transcriptions. The expression of the LacI gene was further suppressed by Cas9-VP64/sgRNA2, and the luciferase reporter gene was, therefore, activated by transcription. In normal bladder epithelial cells, luciferase could not be effectively transcribed and was further silenced by trace amounts of LacI expressed at the background level. After reducing the 3.2 kbp DNA sequence from the traditional gene circuit, this minigene circuit was transfected into cells using a single plasmid vector (the traditional gene circuit requires co-transfection of multiple plasmid vectors). The luciferase expression level of the reporter gene was detected at 48 h after plasmid transfection, and compared with the traditional version of the complex gene circuit. Luciferase expression leakage in normal cells was almost absent in the minigene circuit, while luciferase expression levels in the high malignant subgroup of bladder cancer cells were about 1.5-fold higher than that in the low malignant subgroup (Fig. 1b). The above results suggested that the minigene circuit had a higher sensitivity for recognition of bladder cancer than the traditional gene circuit.

To further analyze the molecular regulatory mechanism for explaining the difference in efficiency, we tested the transcriptional activity of the TERT and UPII promoters in these same cell lines, as well as the expression levels of c-Myc and Get1. The UPII promoter only had a higher transcription activity in low-grade bladder cancer cell lines (Supplementary Fig. 1a). However, the expression levels of both c-Myc and Get1 increased with increases in the malignancy of bladder cancer (Supplementary Fig. 1b). To further clarify the tissue specificity of the minigene circuit, we then confirmed that Get1 had a high expression level in the epithelium of human bladder tissues, while its expression level was low in many other tissues (Supplementary Fig. 2a). The minigene circuit also could not strongly drive the expression of luciferase in several cancer cell lines with other tissue types compared to that in a bladder cancer cell line (T24) (Supplementary Fig. 2b).

### Minigene circuits specifically and efficiently induced apoptosis of bladder cancer cells

**Fig. 2:**
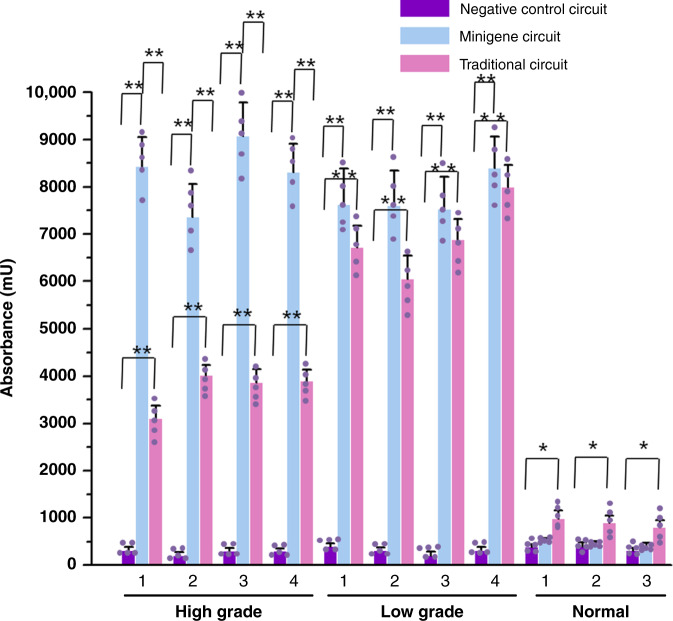
The apoptosis levels of bladder cell lines transfected with either the minigene circuit or the traditional circuit. The level of cell apoptosis was calculated using the Cell Death Detection ELISA assay. Results are shown as the mean ± SD. The minigene circuit without output gene was used as the negative control. Each experiment was performed in triplicate for five independent times. Each error bar indicates the variation (standard deviation) between the means of five independent experiments. The small purple dots represent the mean of each independent experiment. The numerical category label represented the number of the corresponding cell line in each group. mU = absorbance [10^−3^]. ^*^p < 0.05 and ^**^p < 0.01 between the groups using a two-tailed *t*-test. Exact *p-*values for asterisks (from left to right): 0.0002, 0.0056, 0.0043, 0.0005, 0.0041, 0.0061, 0.0001, 0.0047, 0.0052, 0.0002, 0.0045, 0.0058, 0.0006, 0.0008, 0.0005, 0.0009, 0.0005, 0.0007, 0.0003, 0.0004, 0.025, 0.031, and 0.035. Source data are provided as a Source Data file.

We then asked whether the minigene circuit could selectively and effectively induce apoptosis of bladder cancer cells by regulating expression of the proapoptotic human Bax gene, which was used in the AND gate constructed in our previous work^14^. The output was swapped from the luciferase gene to the Bax gene, and the cell-killing ability of the minigene circuit was determined using cell death detection ELISA values relative to the traditional AND gate gene circuit. The minigene circuit selectively increased hBAX expression (Supplementary Fig. 3) and triggered apoptosis in bladder cancer cells of varying degrees of malignancy without affecting normal urothelial cells (Fig. 2). In contrast, although the traditional AND gate gene circuit could significantly induce the apoptosis of both low-grade and high-grade bladder cancer cells, its ability to induce apoptosis of high-grade bladder cancer cells was relatively low compared to that of the minigene circuit. In addition, the traditional gene circuit had a weak toxicity to normal urothelial cells, indicating that the TERT and UPII promoters still had some leakage of activity in normal cells. These results suggested that the minigene AND gate circuit could be used to specifically and effectively kill bladder cancer cells.

### Minigene circuits specifically and efficiently inhibited proliferation of bladder cancer cells

**Fig. 3:**
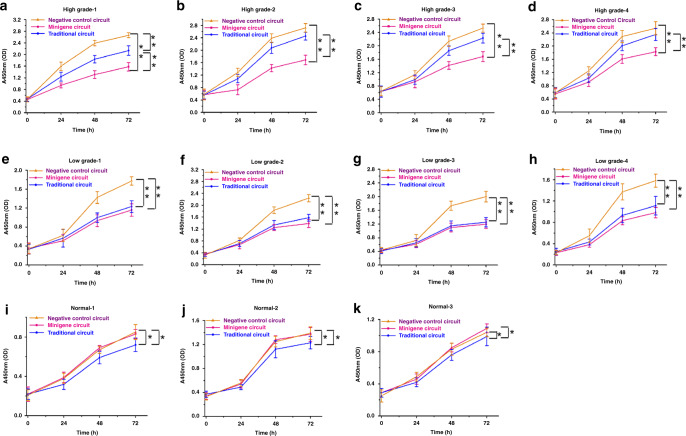
Growth curves of bladder cell lines transfected with either the minigene circuit or the traditional circuit. Proliferations of the transfected cells “high grade-1” (**a**), “high grade-2” (**b**), “high grade-3”(**c**), “high grade-4” (**d**), “low grade-1” (**e**), “low grade-2” (**f**), “low grade-3”(**g**), “low grade-4” (**h**), “normal-1” (**i**), “normal-2” (**j**), and “normal-3” (**k**) were measured using the CCK-8 assay at different time intervals. The minigene circuit without output gene was used as the negative control. The curves of cell proliferations were compared using two-way analysis of variance (ANOVA). The results at each time point are shown as the mean ± SD. Each experiment was independently performed in triplicate five times. Each error bar indicates the variation between the means of five independent experiments. ^*^p < 0.05 and ^**^p < 0.01 between the groups. Exact *p*-values for asterisks (from left to right): 0.0008, 0.0059, 0.0061, 0.0007, 0.0034, 0.0011, 0.0057, 0.0019, 0.0063, 0.0023, 0.0021, 0.0064, 0.0062, 0.0055, 0.0054, 0.0059, 0.0053, 0.034, 0.034, 0.036, 0.036, 0.041, and 0.038. OD optical density. Source data are provided as a Source Data file.

We then further determined the efficiency of the minicircuit design by replacing the luciferase gene with the p21 gene and examined whether the circuit could control cell proliferation by regulating expression of the p21 gene, which suppressed cell proliferation and was also used in the AND gate constructed in our previous work^14^. Cell proliferation was determined at various time points using the CCK-8 assay. Data presented in Fig. 3 and Supplementary Fig. 4 show that the minigene circuit selectively increased p21 expression and strongly suppressed proliferation of bladder cancer cells of varying stages, without affecting normal urothelial cells. The ability of the traditional AND gate gene circuit to inhibit the proliferation of bladder cancer cells with low malignant levels was more significant (Fig. 3e–h), while the inhibitory effect on bladder cancer cells with high malignant levels was relatively low (Fig. 3a–d). To a certain extent, the traditional gene circuit also inhibited the proliferation of normal bladder epithelial cells. These results suggested that the minigene circuit could be used to specifically and strongly inhibit the proliferation of bladder cancer cells.

### Minigene circuits specifically and efficiently suppressed migration of bladder cancer cells

**Fig. 4:**
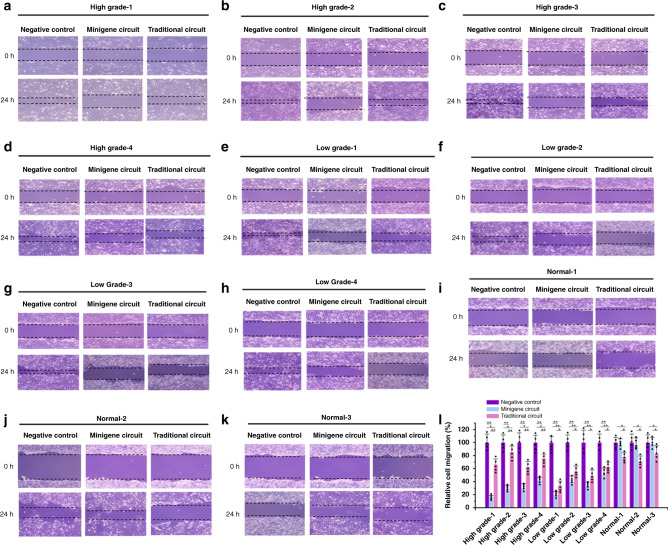
Migrations of bladder cell lines transfected with either the minigene or traditional circuits. Migrations of the transfected cells “high grade-1” (**a**), “high grade-2” (**b**), “high grade-3” (**c**), “high grade-4” (**d**), “low grade-1” (**e**), “low grade-2” (**f**), “low grade-3”(**g**), “low grade-4” (**h**), “normal-1” (**i**), “normal-2” (**j**), and “normal-3” (**k**) were measured using the scratch assay. Data are expressed as the mean ± SD (**l**). The minigene circuit without output gene was used as the negative control. Relative cell migration (%) in each group was determined by the migration distance normalized to that of the negative control group. Each experiment was independently performed in triplicate five times. Each error bar indicates the variation between the means of five independent experiments. The small black dots represent the mean of each independent experiment. ^*^p < 0.05 and ^**^p < 0.01, between the groups, using a two-tailed *t*-test. Exact *p-*values for asterisks (from left to right): 0.0033, 0.027, 0.0087, 0.0042, 0.045, 0.0081, 0.0043, 0.025, 0.0096, 0.0052, 0.031, 0.0093, 0.0036, 0.0045, 0.043, 0.0049, 0.0068, 0.042, 0.0043, 0.0062, 0.044, 0.0054, 0.0074, 0.046, 0.021, 0.033, 0.017, 0.029, 0.024, and 0.036. Source data are provided as a Source Data file.

Next, the output was swapped from the luciferase gene to the E-cadherin gene, which is a strong migration suppressor in cancer cells, and was used in the AND gate constructed in our previous work. Cell migration was determined using a wound-healing assay. Compared with the traditional AND gate gene circuit, the minigene circuit selectively increased E-cadherin expression (Supplementary Fig. 5) and showed a more robust inhibition of migration in bladder cancer cells in different stages (Fig. 4). In addition, the minigene circuit did not present inhibitory effects on bladder epithelial cells, while the traditional AND gate gene circuit had small but significant effects on these cells. These data suggested that the minigene circuit could be used to specifically and strongly inhibit the migration of bladder cancer cells.

### Minigene circuits efficiently reduced in vivo tumor growth and metastasis

**Fig. 5:**
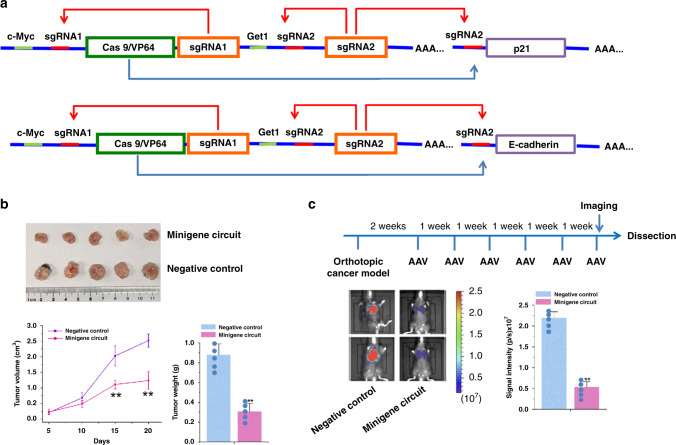
Minigene circuits specifically and efficiently inhibited in vivo tumor growth and metastasis. **a** The design of minigene circuits targeting cellular genes p21 and E-cadherin. Among them, p21 and E-cadherin are located on the recipient cell genome, while other components are located on the AAV vector. **b** The tumor volume was calculated once every 5 days after injecting AAVs. The minigene circuit with sgRNA control that had no targeted intracellular gene was used as the negative control circuit. Tumors treated with the minigene circuit (*n* = 5 animals) targeting p21 were dramatically smaller than those treated with the negative control (*n* = 5 animals). Data are shown as means of tumor weight ± SD. The small blue dots represent the weight of each sample. ^**^p < 0.01, relative to the negative control using the two-tailed *t*-test. Exact *p-*values for asterisks (from left to right): 0.0006, 0.0004, and 0.037. Source data are provided as a Source Data file. **c** Quantification for bioluminescence imaging of a metastatic model. The minigene circuit with sgRNA control that had no targeted intracellular gene was used as the negative control circuit. The luminescent signal intensities for either minigene circuit targeting E-cadherin (*n* = 5 animals) or negative control circuit (*n* = 5 animals) are shown. Only tumors in lung regions were quantified by intraperitoneal D-luciferin administration, and then the signal intensities in lungs were calculated. The small blue dots represent the mean signal intensity of each mouse. ^**^p < 0.01 (*p* = 0.029), relative to the negative control using the two-tailed *t*-test. AAV adeno-associated virus. Source data are provided as a Source Data file.

As the biggest advantage of the minigene circuits is the small capacity and ease of loading with delivery vectors, we determined whether the AAV-minigene circuits could inhibit tumor growth and metastasis in vivo. Minigene circuits targeting intracellular genes (p21 and E-cadherin) were designed, and an all-in-one AAV2 delivery vector, which has been shown to mediate delivery of gene therapy systems to bladder cancer^24^ was constructed. The traditional logical AND gate gene circuits could not be integrated into a single AAV, due to the small packaging capacity of the AAV vectors. One above-mentioned bladder cancer cell line (No.9, Supplementary Table 1) with tumorigenic ability in vivo was chosen and then the cells were inoculated into male nude mice. The human bladder cancer nude mouse transplanted tumor model was established 10 days after inoculation. After direct intratumor injection of AAVs, we found that the tumors treated with the minigene circuits (p21) group were dramatically smaller than those in the negative control group (Fig. 5), and the average tumor weight was significantly lower in the minigene circuits (p21) group compared to the negative control group at the end of the experiment. Meanwhile, compared with the negative control, the minigene circuit strongly increased p21 expression in these tumors (Supplementary Fig. 6a). A progressively more metastatic human bladder cancer cell line (No.16, Supplementary Table 1) was then used to develop an in vivo bladder cancer lung metastasis model. After intravesical infusion of AAVs, the minigene circuit (E-cadherin) significantly suppressed the metastatic effect. Compared with the negative control circuit, the minigene circuit also strongly increased E-cadherin expression in these tumors (Supplementary Fig. 6a). To further investigate the specificity of the minigene circuit in vivo, the E-cadherin expression level in normal tissue surrounding the tumor was also determined. No difference in the expression of E-cadherin between the minigene circuit group and the negative control group was found (Supplementary Fig. 6b). Altogether, these results suggested that the minigene circuit could be used to strongly inhibit the growth and metastasis of bladder cancer cells in vivo.

## Discussion

The related signaling proteins do not alone play a role in cancer development, but instead form a network through interactions^25^. Many previous studies only reported regulation of a small number of target molecules in the signaling pathway, because their technology lacked directional recognition and regulation based on the signaling network^26^. To resolve this problem, several AND gate genetic circuits that sense and process various endogenous signals have been used to recognize and treat cancer. Xie et al.^27^ constructed a multi-input RNAi-based logic circuit for the identification of specific HeLa cancer cells. Nissim et al.^28^ synthesized an RNA-based AND gate that generates combinatorial immunomodulatory outputs only when both synthetic cancer-specific promoters are active. Chung et al.^29^ used modular synthetic proteins to build a compact synthetic pathway that rewires cancer signaling to therapeutic effector release. In addition, the CRISPR-Cas9 technology can also be used to build a logical AND gate that uses tumor-specific promoters as sensors for input signals in target cells, which specifically interfere with bladder cancer cells by controlling the expression of anticancer related genes without affecting normal cells^13,14^. This provides a strategy for targeted and precise intervention in tumors. However, the large size of the CRISPR-Cas9 system made it difficult to package it into a single AAV vector for primary cell and in vivo delivery, and gene editing and regulation mediated by multiple AAV vectors is less effective than that mediated by a single AAV vector^21^.

Natural gene regulatory elements often contain a large number of complex motifs^15^, such as promoters containing binding sites for various transcriptional activation and repressors, and 5′-UTRs containing translation initiation elements, ribosome binding sites, and other complex regulatory sequences. As a result, unknown interactions and crosstalk occur, and artificial logical AND gate gene circuits are highly complex and difficult to predict^18^. Therefore, their sensitivities and specificities for identification of disease cells are often limited.

In the present study, we described improvement and optimization of the previously developed logical AND gate genetic circuit by miniaturizing and simplifying the genetic circuit with CRISPReader technology. The minigene circuits could be delivered into tissue cells through the all-in-one AAV vector, thereby significantly improving the intracellular working efficiency. The minigene circuits significantly induced bladder cancer-specific gene expression, showing robust output gene expression in bladder cancer cells, but with nearly undetectable output expression in normal bladder epithelial cells. They had a higher capability for cancer identification and intervention than the traditional gene circuits in vitro, and showed a strong anticancer effect in vivo. As there are too many transcription factor binding sites for the TERT and UPII promoters used in traditional gene circuits, the activity of the output gene is largely affected by oscillations of different transcription factors in the cells^30^. For example, the UPII promoter was inactive in highly malignant bladder cancer lines, although the Get1 transcription factor was expressed at high levels. This may be because some transcriptional repressors are up-regulated in highly malignant cell lines. In contrast, the minigene circuits were only regulated by c-myc and Get1 transcription factors, and therefore they could respond precisely to the malignant transformation of bladder cancer cells. The above reasons led to a significant difference between the minigene circuits and the traditional gene circuits in the recognition and killing efficiency of bladder cancer.

The minigene circuits offer unique advantages for precision medical research on malignant tumors and other diseases, and can expand design ideas and concepts for gene circuits in synthetic biology.

In conclusion, this study constructed minigene circuits that selectively and robustly mediated gene expression in human bladder cancer cells. It would, therefore, be of great interest to extend these circuits to clinical research because AAV is a commonly used vector for gene therapy.

## Methods

### Ethics statement

The ethical approval board of The First Affiliated Hospital of Shenzhen University approved the use of patient tissue samples that were used to establish the cell lines. Written informed consent was obtained from the patients involved in this study and that the use of human patient material meets MOST guidelines. The RNA samples of human tissues were provided by the Biobank of The First Affiliated Hospital of Shenzhen University and the study on Get1 expression was approved by its Ethical Review Board. The use of 22 RNA samples of human tissues also meets MOST guidelines. The Shenzhen University Ethics Committee approved the use of animal work for this study.

### Cell lines and cell culture

The bladder cancer cell lines and normal urothelial cell lines were established from tumor tissues, and the corresponding adjacent normal tissues were established from different patients. The pathological tumor grade was judged by several experienced clinicians by reading pathological slices. The HEK-293T (embryonic kidney), SW480 (colon cancer), TE-10 (esophagus cancer), 786-O (kidney cancer), HepG2 (liver cancer), A549 (lung cancer), Caov-3 (ovary cancer), Panc-1 (pancreas cancer), SGC-7901 (stomach cancer), and T24 (bladder cancer) cell lines were obtained from American Type Culture Collection (ATCC, Manassas, VA, USA). All cell lines were grown in Dulbecco’s Modified Eagle’s Medium (Catalog No. 12320032, DMEM, Invitrogen, Carlsbad, CA, USA) supplemented with 10% fetal bovine serum in the presence of 5% CO_2_. During the current study, cell cultures were free of Mycoplasma contamination as detected by 16S rRNA-based Mycoplasma group-specific PCR.

### Construction of AND gate minigene circuits

The original sgRNAs were designed using the online design tool “CRISPR-ERA Version 1.2” (http://CRISPR-ERA.stanford.edu). The complementary DNA (cDNA) sequences for sgRNA binding regions were synthesized, and inserted into corresponding vectors digested with restriction endonucleases. The plasmids, cMyc-Get1-Cas9/VP64-sgRNA-lacI-output, expressing the AND gate minigene circuits were then constructed based on the backbone plasmids, which have been used in our previous reports^11,12^. All vectors were transformed into One Shot TOP10 chemically competent *E. coli* cells (Catalog No. C404010, Invitrogen, Carlsbad, USA). After overnight incubation at 37 °C on LB plates containing 100 μg/mL ampicillin, recombinant colonies were selected and plasmid DNA was extracted from confirmed bacterial colonies with a Qiagen Plasmid Mini Kit (Catalog No. 27104, Qiagen, Hilden, Germany) according to the manufacturer’s instructions. The plasmid DNA were identified using molecular amplification and electrophoresis, and then confirmed with Sanger sequencing. The sequence information is shown in Supplementary Table 2. Maps of plasmids expressing minigene circuits are shown in Supplementary Fig. 7.

### Cell transfection

For DNA transient transfections, bladder cancer cells and normal cells were cultured in 6-well plates (1.5 × 10^5^ cells/well) until they reached 70% confluency, and then were transfected with 2.5 μg plasmids using Lipofectamine 3000 (Catalog No. L3000015, Invitrogen, Carlsbad, CA, USA) according to the manufacturer’s protocol, followed by collection of transfected cells after at least 48 h.

### Luciferase assay

The PGL3-TK-Fluc vector (200 ng per transfection)^14^ in which Fluc luciferase is constitutively expressed was used as the transfection control in the luciferase assay. Luciferase activity was measured in a 1.5 mL Eppendorf tube using the Dual-Luciferases Reporter Assay kit (E1980; Promega, Madison, WI, USA) according to manufacturer’s protocols at 48 h after DNA transfection. The relative Renilla luciferase activity was calculated as a value normalized to that of the firefly luciferase activity. The assays were performed in triplicate and the experiments were repeated three times.

### RNA extraction and real-time quantitative PCR

Tissue samples were stored in RNALater (Catalog No. AM7020, Thermo Fisher Scientific, Waltham, Ma, USA), and total RNAs were isolated from cells at 48 h after DNA transfection or mouse tissues using TRIzol reagent (Catalog No. 15596026, Invitrogen, Carlsbad, CA, USA) according to the manufacturer’s protocol. The concentration and purity of total RNA were measured using UV spectrophotometric analysis at 260 nm. The cDNAs were synthesized using a Revertra Ace qPCR RT Kit (Catalog No. FSQ-101, Toyobo, Osaka, Japan). Real-time PCR was conducted using real-time PCR Master Mix (Catalog No. FSQ-201, Toyobo, Osaka, Japan). Glyceraldehyde 3-phosphate dehydrogenase (GAPDH) was used as the endogenous control. The PCR mixtures were prepared according to the manufacturer’s protocol and amplification was performed under PCR conditions of 40 cycles of 15 s at 95 °C, 20 s at 55 °C, and 30 s at 70 °C on a ABI PRISM 7300 Fluorescent Quantitative PCR System (Applied Biosystems, Foster City, CA, USA). Primer sequences are listed in Supplementary Table 3. Expression-fold changes were calculated using the 2^−△△ct^ method^31^.

### Cell apoptosis assay

According to the protocol supplied by the manufacturer, cell apoptosis was determined by quantifying histone-complexed DNA fragments (nucleosomes) in the cytoplasm using a cell death detection ELISA (Catalog No.11774425001, Roche Applied Science, Indianapolis, IN, USA) at 48 h after DNA transfection. The absorbance, which was proportional to the amount of nucleosomes released into the cytoplasm, was measured at 405 nm using a microplate reader (Bio-Rad, Hercules, CA, USA). The experiment for each sample was independently repeated at least three times.

### Cell proliferation assay

Cell proliferation assays were conducted using the Cell Counting Kit-8 (CCK-8) (Catalog No. C0038, Beyotime, Shanghai, China) according to the supplier’s instructions. One-hundred microliters of cell suspension (5 × 10^3^ cells) was added onto each well of a 96-well plate. At 0, 24, 48, or 72 h time points, 10 μL of CCK-8 reagent was added to each well of the 96-well plate and the cells were incubated for 1 h. Absorbance was read at a wavelength of 450 nm by using a microplate reader (Bio-Rad, Hercules, CA, USA). The assay was independently conducted at least three times.

### Cell migration assay

Cell migration was determined using the wound-healing assay. Briefly, the cells were seeded in 12-well plates at equal densities and grown to 80% confluency. Artificial gaps were generated using a sterile 200-μL pipette tip. Areas of the wound were marked and photographed at 0 or 24 h after wound generation with a digital camera system. Researchers were blinded for evaluating cell migration, and the HMIAS-2000 software program (Qianping Image Technology Co., Ltd., Wuhan, China) was used to conduct automated analysis of cell migration distance (mm). Each experiment was repeated at least three times.

### Adeno-associated virus (AAV) packaging, purification, and titer detection

The pAAV packaging DNA construct, pHelper construct, and pAAV construct were co-transfected into HEK-293T cells using Lipofectamine 3000. The culture supernatants were collected at 48 h after transfection, concentrated, and used as virus stocks for the following AAV infection experiment. The AAV titer was calculated by qPCR using 2× EvaGreen Master Mix (Catalog No. BG10401S, Syngentech, Beijing, China).

### Tumor xenografts

The tumor xenograft assay and animal protocols were approved by the Shenzhen University Ethics Committee, and the study is compliant with the “Guidance of the Ministry of Science and Technology (MOST) for the Review and Approval of Human Genetic Resources.” The female BALB/c nude mice aged 4–5 weeks were purchased from Guangdong Medical Laboratory Animal Center (Guangzhou, China) and grown under standard laboratory conditions (temperature 24–26 °C, 12–12 hour dark–light circle and humidity 45–65%) and fed with a laboratory diet and water. The mice were randomly divided into an experimental or control group (five mice per group). A total of 5 × 10^7^ bladder cancer cells (No.9, Supplementary Table 1) were hypodermically injected on the back of BALB/c-Nude mice, and the mice were then treated with AAVs after 10 days of inoculation. Intratumoral injection of AAV (100 μL, 2 × 10^11^ vp/mL) was conducted. The volumes of tumors were calculated every 5 days after AAV injection using the formula: *V* = *L* × *W*^2^/2, where *L* is the length and *W* is the width of the tumor. *L* and *W* were measured by a digital caliper. The mice were sacrificed and the tumors were removed and weighted at the end of the experiment (20 days after injecting AAVs). The tumor tissues were stored in RNALater (Catalog No. AM7020, Thermo Fisher Scientific, Waltham, Ma, USA) and frozen in liquid nitrogen after resection, and TRIzol reagent was used to extract total RNA for examination of targeted gene expression.

### Mouse metastasis model experiment

The mouse metastasis model experiment and animal protocols were approved by the Shenzhen University Ethics Committee, and the study is compliant with the “Guidance of the Ministry of Science and Technology (MOST) for the Review and Approval of Human Genetic Resources”. Mice purchased from the same source and cultured under the same conditions as the tumor xenograft assay described above were divided into an experimental group and a control group, with five mice in each group. A total of 5 × 10^5^ bladder cancer cells (No.16, Supplementary Table 1) encoding luciferase, in 50 μL solution, were implanted orthotopically into the bladder wall using a 30 gauge needle. On day 14 when the tumors size reached around 500 mm^3^, AAV (100 μL, 2 × 10^11^ vp/mL) was infused into the bladder once a week for a total of six times. At each time, the urethra was tied with nylon to retain the AAV vector in the bladder and released after 4 h. Five weeks later, immediately after the final AAV administration, the mice were anaesthetized with isoflurane, and D-luciferin sodium salt (150 mg/kg) was injected intraperitoneally. Then, the cancer cells were detected with an in vivo imaging system (Xenogen IVIS; PerkinElmer, Waltham, MA, USA). The total flux in photons per second was calculated and reported for each mouse’s lung region using Living Image 4.3.1 (PerkinElmer/Caliper). The animals were sacrificed 3 days after the last treatment, and the bladder tissues were excised. The tumor tissues and pair-matched normal tissues were stored in RNALater (Catalog No. AM7020, Thermo Fisher Scientific, Waltham, Ma, USA) and frozen in liquid nitrogen after resection, and TRIzol reagent was used to extract total RNA for examination of targeted gene expression.

### Statistical analyses

All the data were collected and listed using Excel 2007 (Microsoft Corp., Redmond, WA, USA). Data were expressed as the mean ± standard deviation (SD). Significance tests were performed using SPSS statistical software for Windows, version 17.0 (SPSS, Chicago, IL, USA). Statistical significance, determined using Student’s *t*-test or analysis of variance (ANOVA), was assigned at *p* < 0.05.

### Reporting summary

Further information on research design is available in the Nature Research Reporting Summary linked to this article.

## Supplementary information

Supplementary Information

Reporting Summary

## Data Availability

The data generated or analyzed during this study are within the Article, Supplementary Information files, or available from the corresponding author upon reasonable request. Source data are provided with this paper.

## Source data

Source Data
