# Supplementary material for: Synthesizing AND gate minigene circuits based on CRISPReader for identification of bladder cancer cells
Source: Nat Commun. 2020 Oct 30;11:5486. doi: 10.1038/s41467-020-19314-7 (PMC7599332; doi:10.1038/s41467-020-19314-7)
Supplement: Supplementary file 1 — Supplementary Information [file 41467_2020_19314_MOESM1_ESM.pdf]

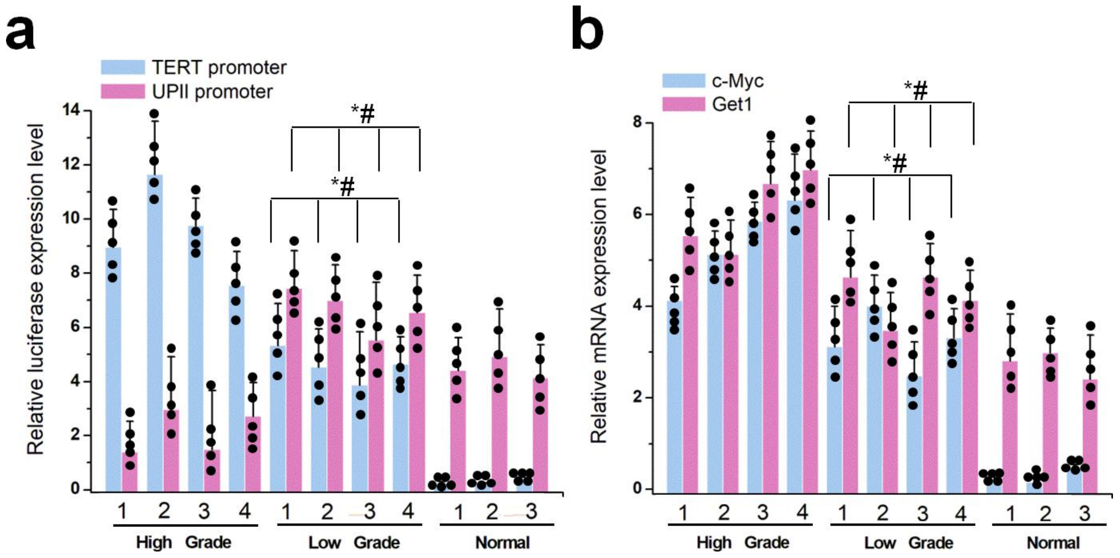

Supplementary Figure 1. The expression levels of luciferase and transcription factors in different cell lines. Data are shown as mean ± SD. Each experiment was performed in triplicate for five independent times. Each error bar indicates the variation between the means of five independent experiments. The small black dots represent the mean of each independent experiment. * $p<0.01$ (From left to right: 0.0032,0.0029,0.0034 and 0.0041), high grade vs. low grade using two-way ANOVA. $^{\#}p<0.01$ (From left to right: 0.0011,0.0088, 0.0013 and 0.0086), low grade vs. Normal using two-way ANOVA. Source data are provided as a Source Data file. (**a**) Relative luciferase activities were determined as the ratios between Rluc and Fluc values. (**b**) The relative expression levels of c-Myc and Get1 were determined by qRT-PCR. The relative expression level was normalized to GAPDH expression.

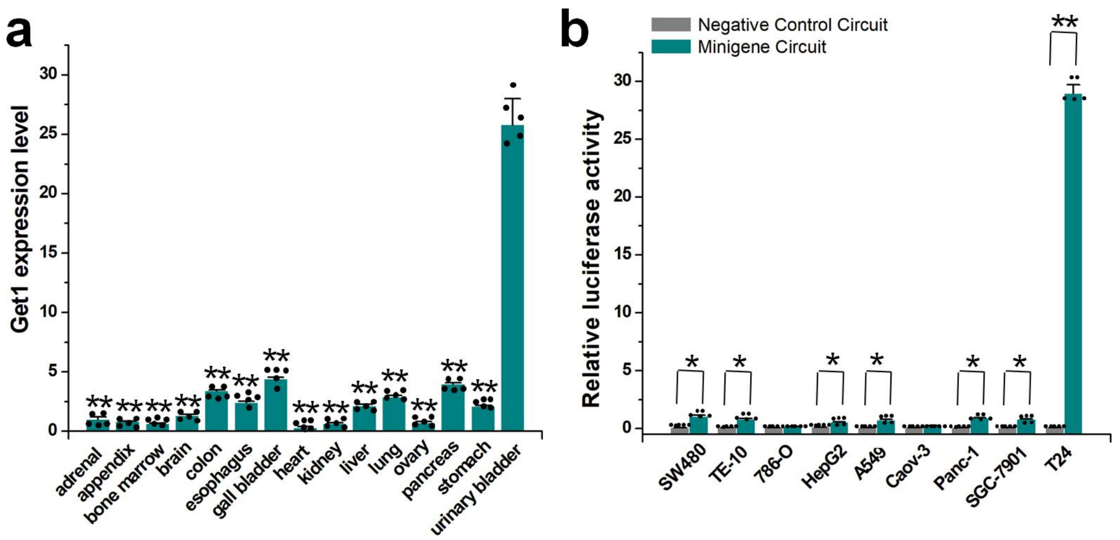

Supplementary Figure 2. Determination of the tissue specificity of the minigene circuit. Data are shown as mean ± SD. Each experiment was performed in triplicate for five independent times. Each error bar indicates the variation between the means of five independent experiments. The small black dots represent the mean of each independent experiment. Source data are provided as a Source Data file. (**a**) Expression of Get1 in various types of human tissues. The relative expression levels of Get1 mRNA were determined by qRT-PCR. The relative expression level (units are arbitrary) was normalized to GAPDH expression. **, p value < 0.01, relative to the bladder tissue by two-tailed *t* test. Exact p values for asterisks (from left to right):0.0013,0.0011,0.0009,0.0016,0.0025,0.0022,0.0031,0.0003,0.0005,0.0022, 0.0024,0.0006,0.0024 and 0.0021. (**b**) The luciferase level of cancer cell lines transfected with the minigene circuit or the negative control circuit. All transient transfections were performed in the presence of PGL3-TK-Fluc vector. Relative luciferase activities were determined as the ratios between Rluc and Fluc values. The minigene circuit without output gene was used as the negative control. *, p value < 0.05, and **, p value < 0.01, relative to the negative control by two-tailed *t* test. Exact p values for asterisks (from left to right): 0.023,0.037,0.028,0.021,0.019.0.018 and 0.0004.

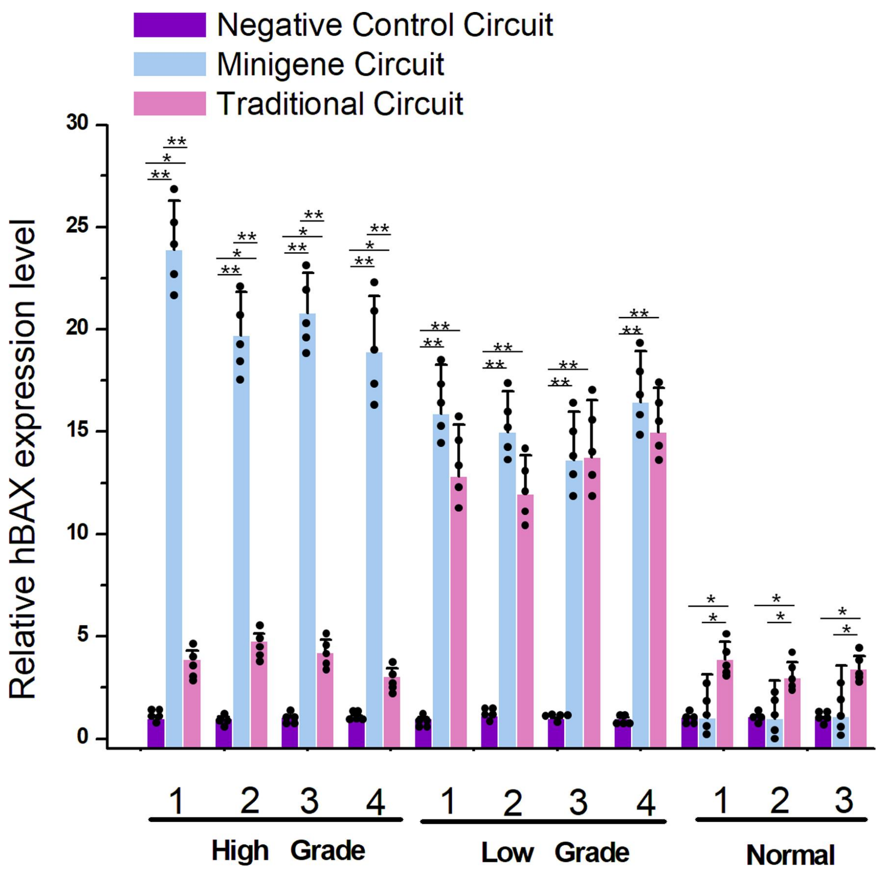

Supplementary Figure 3. The minigene circuit selectively increased *hBAX* expression in bladder cancer cells. The relative expression level of *hBAX* was determined by qRT-PCR. The minigene circuit without output gene was used as the negative control. The relative expression level was normalized to GAPDH expression. Data are shown as mean ± SD. Each experiment was performed in triplicate for five independent times. Each error bar indicates the variation between the means of five independent experiments. The small black dots represent the mean of each independent experiment. *, p value < 0.05, and **, p value < 0.01, by two-tailed *t* test. Exact p values for asterisks (from left to right): 0.0005,0.031,0.0017,0.0008,0.029, 0.0027,0.0007,0.032,0.0025,0.0009,0.036,0.0031,0.0016,0.0041,0.0018,0.0044,0.002 1,0.0038,0.0014,0.0029,0.013,0.033,0.019,0.023,0.018 and 0.025. Source data are provided as a Source Data file.

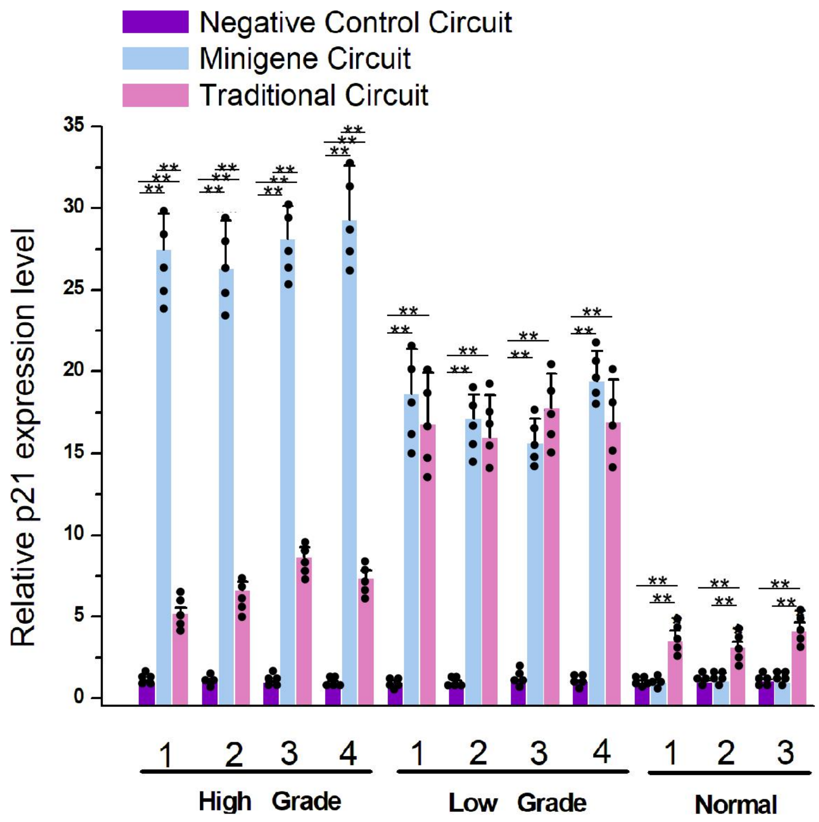

Supplementary Figure 4. The minigene circuit selectively increased *p21* expression in bladder cancer cells. The relative expression level of *p21* was determined by qRT-PCR. The minigene circuit without output gene was used as the negative control. The relative expression level was normalized to GAPDH expression. Data are shown as mean ± SD. Each experiment was performed in triplicate for five independent times. Each error bar indicates the variation between the means of five independent experiments. The small black dots represent the mean of each independent experiment.*, p value < 0.05, and **, p value < 0.01, by two-tailed *t* test. Exact p values for asterisks (from left to right): 0.0007,0.031,0.0017,0.0008,0.029, 0.0025,0.0005,0.027,0.0027,0.0003,0.028,0.0021,0.0016,0.0041,0.0018,0.0044,0.002 2,0.0029,0.0013,0.0035,0.018,0.024,0.021,0.031,0.016 and 0.022. Source data are provided as a Source Data file.

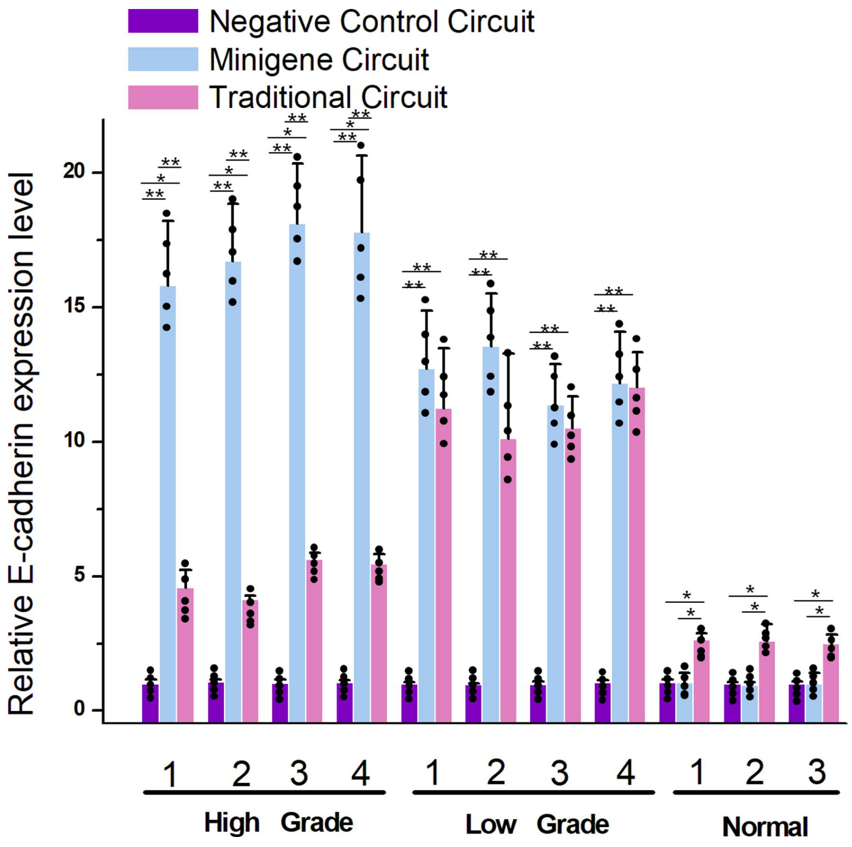

Supplementary Figure 5. The minigene circuit selectively increased *E-cadherin* expression in bladder cancer cells. The relative expression level of *E-cadherin* was determined by qRT-PCR. The minigene circuit without output gene was used as the negative control. The relative expression level was normalized to GAPDH expression. Data are shown as mean ± SD. Each experiment was performed in triplicate for five independent times. Each error bar indicates the variation between the means of five independent experiments. The small black dots represent the mean of each independent experiment. *, p value < 0.05, and **, p value < 0.01, by two-tailed *t* test. Exact p values for asterisks (from left to right): 0.0009,0.027,0.0025,0.0006,0.033, 0.0022,0.0004,0.024,0.0015,0.0005,0.026,0.0018,0.0018,0.0042,0.0016,0.0043,0.002 1,0.0038,0.0019,0.0027,0.013,0.031,0.014,0.033,0.016 and 0.034. Source data are provided as a Source Data file.

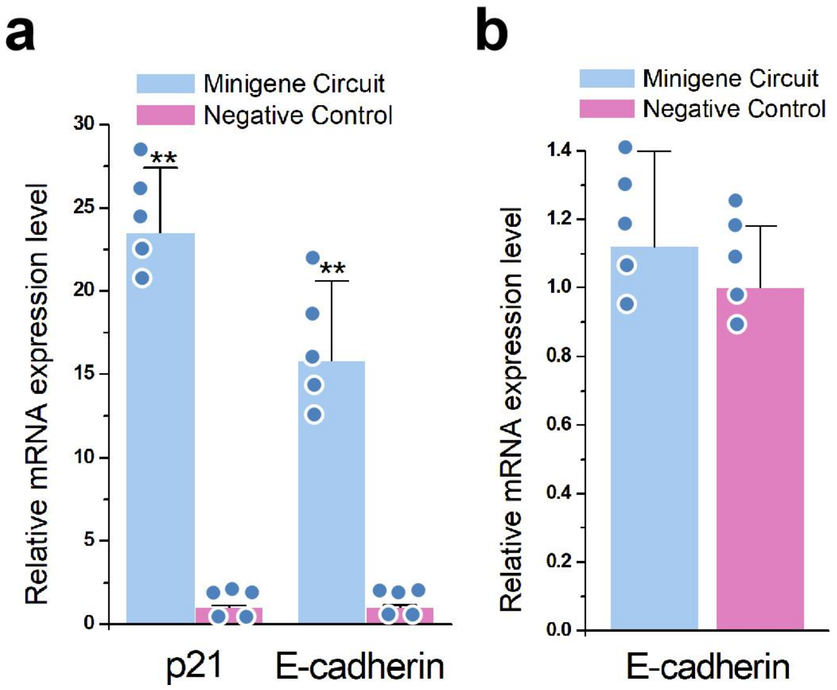

Supplementary Figure 6. The minigene circuit specifically activated *p21* and *E-cadherin* expression in bladder cancer tissues. The minigene circuit with sgRNA control that has no targeted intracellular gene was used as the negative control circuit. The relative expression level was normalized to GAPDH expression. Data are shown as mean ± SD. Each experiment was performed in triplicate for five independent times. Each error bar indicates the standard deviation. The small blue dots represent the mean of five independent experiments in each mouse. (**a**) The relative expression levels of *p21* and *E-cadherin* in bladder cancer tissues (n=5) were determined by qRT-PCR. **, p value < 0.01, relative to the negative control by two-tailed *t* test. Exact p values for asterisks (from left to right): 0.0023 and 0.0058.(**b**) The relative expression level of *E-cadherin* in normal tissue (n=5) surrounding tumor was determined by qRT-PCR. Source data are provided as a Source Data file.

a

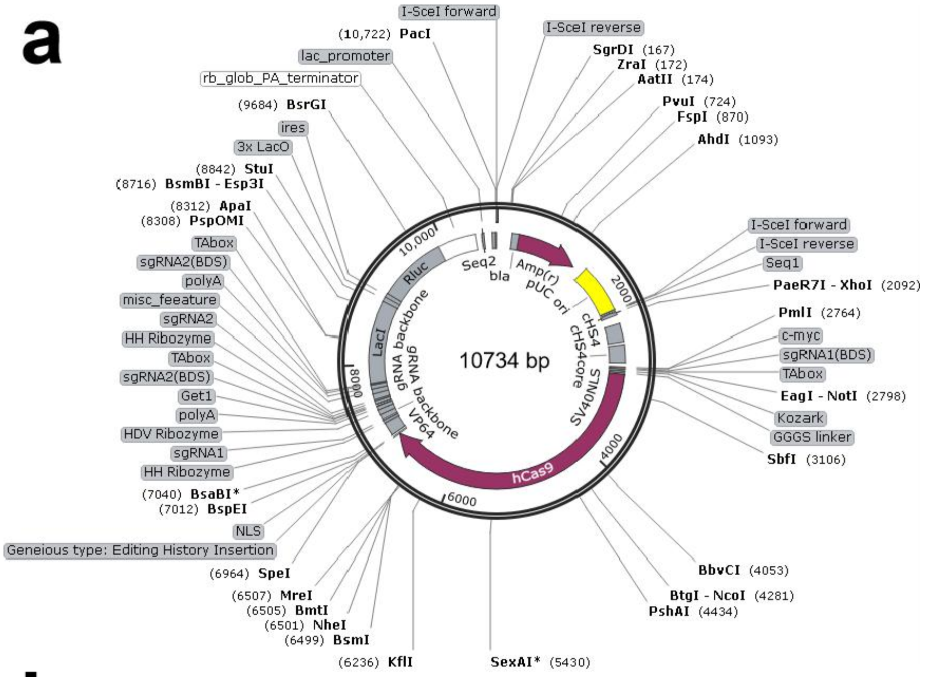

b

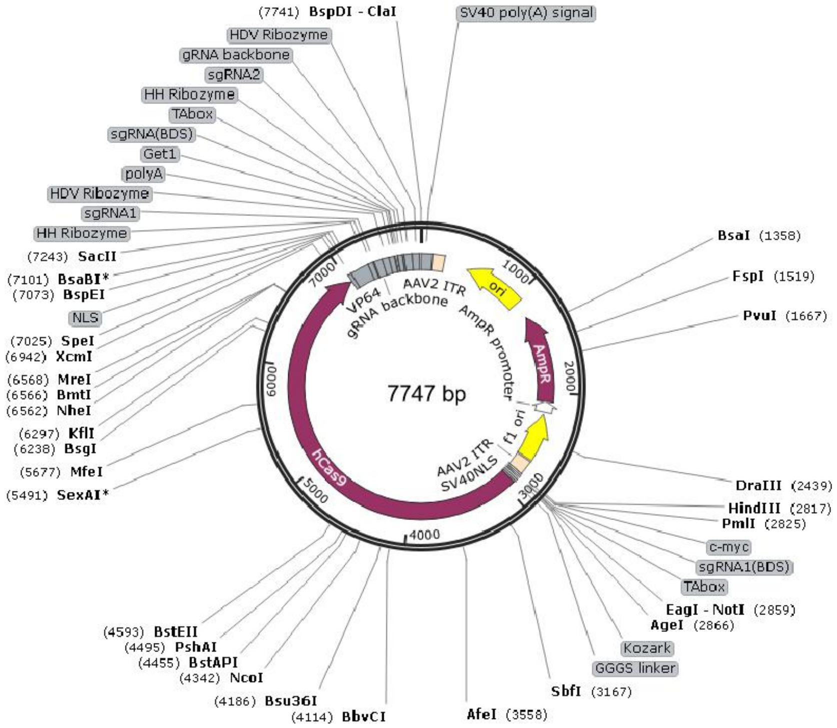

Supplementary Figure 7. Maps of plasmids used in this study. (**a**) The plasmid of the minigene circuit used in cell experiments. The representative figure specifically showed the minigene circuit expressing the luciferase reporter, which was used in figure-1. (**b**) The plasmid of the minigene circuit used in animal experiments, which could be used for packaging AAVs.

Supplementary Table 1. Clinical features of patients with bladder cancer.

No.	Sex	Age	Grade	Stage	Surgery	No.	Sex	Age	Grade	Stage	Surgery
1	M	58	Low	T1N0M0	Partial	11	M	61	High	T3N0M0	Radical
2	M	62	Low	T1N0M0	Partial	12	F	62	High	T2N0M0	Radical
3	M	57	Low	T1N0M0	Partial	13	M	54	High	T2N0M0	Radical
4	F	65	Low	T1N0M0	Partial	14	M	56	High	T3N0M0	Radical
5	M	61	Low	T1N0M0	Partial	15	M	53	High	T3N0M0	Radical
6	M	60	Low	T1N0M0	Partial	16	M	55	High	T3N0M0	Radical
7	M	55	Low	T1N0M0	Partial	17	M	51	High	T3N0M0	Radical
8	F	63	Low	T1N0M0	Partial	18	M	55	High	T2N0M0	Radical
9	M	47	Low	T1N0M0	Partial	19	M	52	High	T2N0M0	Radical
10	M	58	Low	T1N0M0	Partial	20	M	53	High	T3N0M0	Radical

No., patient number. M, male. F, female. Age, years old. Grade, the 2004 WHO classification. Stage, AJCC TNM classification. Radical, radical cystectomy. Partial, partial cystectomy.

Supplementary Table 2. The cDNA sequences of the engineered elements.

Names	Sequences
sgRNA-1 (spacer) for Figs.1 and 5	GTGATAGAGAACGT
sgRNA-1 binding sequence for Figs.1 and 5	GTGATAGAGAACGTAGG
sgRNA-2-*LacI* (spacer) for Fig.1	GCCACGTTTCTGCGAAAACG
sgRNA-2 binding sequence for Fig.1	TTTCTGCGAAAACGCGG
sgRNA-2-*P21* (spacer) for Fig.5	CCTTGTGGGCCTCC
sgRNA-2-*P21* binding sequence for Fig.5	CCTTGTGGGCCTCCAGG
sgRNA-2-*E-cadherin* (spacer) for Fig.5	GCCTCGCATAGACG
sgRNA-2-*E-cadherin* binding sequence for Fig.5	GCCTCGCATAGACGAGG
sgRNA control (spacer) for Fig.5	GTACGTTCTCTATCACTGATA
Get1 binding sequence for Figs.1 and 5	AACCTGTC
c-Myc binding sequence for Figs.1 and 5	CACGTGGGAAG
TATA box for Figs.1 and 5	TATATAA
3X LacO for Fig.1	GAATTGTGAGCGCTCACAAT TGAATTGTGAGCGCTCACAA TTGAATTGTGAGCGCTCACA ATT
5’ processing ribozyme for Figs.1 and 5	CTGATGAGTCCGTGAGGACG AAATC
3’ processing ribozyme for Figs.1 and 5	ACCGGAGTCGGGTCTGATGA GTCCGTGAGGACGAAA
Synthetic poly (A) for Figs.1 and 5	AATAAAATATCTTTATTTTCAT TACATCTGTGTGTTGGTTTTT TGTGTG

Note: Except for the sgRNA-2-*lacI*, the spacer length of designed sgRNAs was only 14 nt, which ensures that Cas9-VP64 only regulated transcription. The binding region of the sgRNA-2 -*lacI* upstream of the TATA box was also only 14 nt.

Supplementary Table 3. Primer sequences used in real-time quantitative PCR.

Names	Sequences
c-Myc-F	TGCTCCATGAGGAGACACC
c-Myc -R	CTTTTCCACAGAAACAACATCG
Get1-F	TCCACAGTCAACATGATGGACG
Get1-R	CTGTCCGAGCTTTCACATGGG
hBAX-F	CCCGAGAGGTCTTTTTCCGAG
hBAX -R	CCAGCCCATGATGGTTCTGAT
p21-F	TGTCCGTCAGAACCCATGC
p21-R	AAAGTCGAAGTTCCATCGCTC
E-cadherin -F	CGAGAGCTACACGTTCACGG
E-cadherin -R	GGGTGTCGAGGGAAAAATAGG
GAPDH-F	CGCTCTCTGCTCCTCCTGTTC
GAPDH-R	ATCCGTTGACTCCGACCTTCAC
