# Supplementary material for: Synthesizing AND gate minigene circuits based on CRISPReader for identification of bladder cancer cells
Source: Nat Commun. 2020 Oct 30;11:5486. doi: 10.1038/s41467-020-19314-7 (PMC7599332; doi:10.1038/s41467-020-19314-7)
Supplement: Supplementary file 2 — Reporting Summary [file 41467_2020_19314_MOESM2_ESM.pdf]

nature research

Corresponding author(s): Yuchen Liu

Last updated by author(s): Oct 6, 2020

Reporting Summary

Nature Research wishes to improve the reproducibility of the work that we publish. This form provides structure for consistency and transparency in reporting. For further information on Nature Research policies, see our Editorial Policies and the Editorial Policy Checklist.

Statistics

For all statistical analyses, confirm that the following items are present in the figure legend, table legend, main text, or Methods section.

n/a	Confirmed	
☐	☒	The exact sample size (*n*) for each experimental group/condition, given as a discrete number and unit of measurement
☐	☒	A statement on whether measurements were taken from distinct samples or whether the same sample was measured repeatedly
☐	☒	The statistical test(s) used AND whether they are one- or two-sided<br>*Only common tests should be described solely by name; describe more complex techniques in the Methods section.*
☐	☒	A description of all covariates tested
☒	☐	A description of any assumptions or corrections, such as tests of normality and adjustment for multiple comparisons
☐	☒	A full description of the statistical parameters including central tendency (e.g. means) or other basic estimates (e.g. regression coefficient) AND variation (e.g. standard deviation) or associated estimates of uncertainty (e.g. confidence intervals)
☐	☒	For null hypothesis testing, the test statistic (e.g. *F*, *t*, *r*) with confidence intervals, effect sizes, degrees of freedom and *P* value noted<br>*Give P values as exact values whenever suitable.*
☒	☐	For Bayesian analysis, information on the choice of priors and Markov chain Monte Carlo settings
☒	☐	For hierarchical and complex designs, identification of the appropriate level for tests and full reporting of outcomes
☒	☐	Estimates of effect sizes (e.g. Cohen's *d*, Pearson's *r*), indicating how they were calculated

Our web collection on statistics for biologists contains articles on many of the points above.

Software and code

Policy information about availability of computer code

Data collection	Microsoft Excel 2007 (Microsoft Corp., Redmond, WA, USA).
Data analysis	SPSS statistical software for Windows, version 17.0 (SPSS, Chicago, IL, USA); CRISPR-ERA, version 1.2 (http://CRISPR-ERA.stanford.edu); HMIAS-2000 software program (Qianping Image Technology Co., Ltd., Wuhan, China) ;in vivo imaging system (Xenogen IVIS; PerkinElmer, Waltham, MA, USA).

For manuscripts utilizing custom algorithms or software that are central to the research but not yet described in published literature, software must be made available to editors and reviewers. We strongly encourage code deposition in a community repository (e.g. GitHub). See the Nature Research guidelines for submitting code & software for further information.

Data

Policy information about availability of data

All manuscripts must include a data availability statement. This statement should provide the following information, where applicable:

- Accession codes, unique identifiers, or web links for publicly available datasets
- A list of figures that have associated raw data
- A description of any restrictions on data availability

The data generated or analyzed during this study are within the Article, Supplementary Information files, or available from the corresponding author upon reasonable request. Source data are provided with this paper.

Field-specific reporting

Please select the one below that is the best fit for your research. If you are not sure, read the appropriate sections before making your selection.

- [x] Life sciences
- [ ] Behavioural & social sciences
- [ ] Ecological, evolutionary & environmental sciences

For a reference copy of the document with all sections, see nature.com/documents/nr-reporting-summary-flat.pdf

Life sciences study design

All studies must disclose on these points even when the disclosure is negative.

Sample size	No statistical methods were used to predetermine sample size. Five independent experiments were performed in this study, which was sufficient to perform statistical test and calculate P value in the field.
Data exclusions	No data were excluded.
Replication	The experimental findings in all figures were reproduced successfully.
Randomization	Samples were not randomized as the sample size was not large.
Blinding	The investigators were blinded to allocation during the experiments and outcome assessment.

Reporting for specific materials, systems and methods

We require information from authors about some types of materials, experimental systems and methods used in many studies. Here, indicate whether each material, system or method listed is relevant to your study. If you are not sure if a list item applies to your research, read the appropriate section before selecting a response.

Materials & experimental systems

n/a	Involved in the study	
x		Antibodies
	x	Eukaryotic cell lines
x		Palaeontology and archaeology
	x	Animals and other organisms
	x	Human research participants
x		Clinical data
x		Dual use research of concern

Methods

n/a	Involved in the study	
x		ChIP-seq
x		Flow cytometry
x		MRI-based neuroimaging

Eukaryotic cell lines

Policy information about cell lines

Cell line source(s)	The bladder cancer cell lines and normal urothelial cell lines were established from tumor tissues, and the corresponding adjacent normal tissues were established from different patients. The HEK-293T (embryonic kidney), SW480 (colon cancer), TE-10 (esophagus cancer), 786-O (kidney cancer), HepG2 (liver cancer), A549 (lung cancer), Caov-3 (ovary cancer), Panc-1 (pancreas cancer), SGC-7901 (stomach cancer) and T24 (bladder cancer) cell lines were obtained from American Type Culture Collection (ATCC, Manassas, VA, USA).
Authentication	No cell lines have were authenticated.
Mycoplasma contamination	All cell lines have been tested for mycoplasma contamination free.
Commonly misidentified lines (See ICLAC register)	No commonly misidentified cell lines were used.

Animals and other organisms

Policy information about studies involving animals; ARRIVE guidelines recommended for reporting animal research

Laboratory animals	The female BALB/c nude mice aged 4–5 weeks were purchased from Guangdong Medical Laboratory Animal Center (Guangzhou, China) and grown under standard laboratory conditions (temperature 24-26 °C, 12-12 hour dark-light circle and humidity 45-65%) and fed with a laboratory diet and water.
Wild animals	No wild animals were used in this study.

Field-collected samples	No field-collected samples were used in this study.
Ethics oversight	The tumor xenograft assay and animal protocols were approved by the Shenzhen University Ethics Committee.

Note that full information on the approval of the study protocol must also be provided in the manuscript.

Human research participants

Policy information about studies involving human research participants

Population characteristics	Bladder cancer patients and their clinical features were shown in Supplementary Table 1.
Recruitment	The patients were undergoing surgical treatment in the hospital, and the postoperative pathological results were confirmed by at least 2 experienced pathologists. Therefore, there is no obvious bias.
Ethics oversight	the Ethics Committee of the 1st Affiliated Hospital of Shenzhen University. Informed consent was obtained from the patients involved in this study.

Note that full information on the approval of the study protocol must also be provided in the manuscript.
